# Health-related quality of life and life satisfaction in colorectal cancer survivors: trajectories of adjustment

**DOI:** 10.1186/1477-7525-11-46

**Published:** 2013-03-14

**Authors:** Jeff Dunn, Shu Kay Ng, William Breitbart, Joanne Aitken, Pip Youl, Peter D Baade, Suzanne K Chambers

**Affiliations:** 1 Griffith Health Institute, Griffith University, Queensland, Australia; 2 Cancer Council Queensland, Queensland, Australia; 3 School of Social Science, University of Queensland, Queensland, Australia; 4 Department of Psychiatry and Behavioral Sciences, Memorial Sloane-Kettering Cancer Center, New York, USA; 5 School of Public Health, Queensland University of Technology, Queensland, Australia; 6 Health and Wellness Institute, Edith Cowan University, Perth, Australia; 7 Centre for Clinical Research, University of Queensland, Queensland, Australia

**Keywords:** Longitudinal, Cancer, Survivorship, Quality of life, Colorectal

## Abstract

**Background:**

This longitudinal study describes the five year trajectories of health-related quality of life (HR-QOL) and life satisfaction in long term colorectal cancer survivors.

**Patients and methods:**

A population-based sample of 1966 colorectal cancer survivors were surveyed at six time points from five months to five years post-diagnosis. Predictor variables were: socio-demographic variables, optimism; cancer threat appraisal; perceived social support. Quality of life was assessed with the Functional Assessment of Cancer Therapy-Colorectal (HR-QOL); and the Satisfaction with Life Scale. Growth mixture models were applied to identify trajectory classes and their predictors.

**Results:**

Distinct adjustment trajectories were identified for HR-QOL and life satisfaction. Lower optimism, poorer social support, a more negative cognitive appraisal, and younger age were associated with poorer life satisfaction, while survivors with less than 8 years of education had higher life satisfaction. This pattern was similar for overall HR-QOL except that educational level was not a significant predictor and later stage disease and female gender emerged as related to poorer outcomes. One in five survivors reported poorer constant HR-QOL (19.2%) and a small group had poor life satisfaction (7.2%); 26.2% reported constant high HR-QOL and 48.8% had high constant life satisfaction. Socioeconomic disadvantage and remoteness of residence uniquely predicted poorer outcomes in the colorectal cancer specific HR-QOL sub domain.

**Conclusion:**

Although HR-QOL and subjective cognitive QOL share similar antecedents their trajectory patterns suggested they are distinct adjustment outcomes; with life satisfaction emerging as temporally stable phenomenon. Unique patterns of risk support suggest the need to account for heterogeneity in adjustment in longitudinal QOL studies with cancer survivors.

## Background

With nearly three quarters of a million people diagnosed in 2008, colorectal cancer is estimated to be the most common incident cancer in the developed world [1]. In 2008 there were an estimated 3.3 million people living with a diagnosis of colorectal cancer within the previous 5 years [1]. Although there has been a decrease in incidence rates in Australia [2], as in the United States [3], the numbers of people diagnosed with colorectal cancer (CRC) each year continue to increase [2]. Despite having one of the highest incidence rates in the world [1], survival outcomes for Australians diagnosed with colorectal cancer are relatively high compared to other countries [4]. Survival expectations increase the longer they survive, so that CRC cancer patients who have survived seven years after diagnosis can anticipate similar 5-year survival outcomes to the general population [5]. The high prevalence of this disease brings into sharp focus the issue of how colorectal cancer influences long term quality of life.

A recent systematic review of quality of life (QOL) outcomes for long term colorectal cancer survivors concluded that long term QOL overall is good for these patients, but noted that research to date was limited by being largely cross sectional in design [6]. Predictors of poorer outcomes included: younger age, lower income and a smaller social network. A longitudinal study of CRC patients over a ten year period found that CRC survivors reported stable or improved QOL over the first three years from diagnosis, however from three to ten years function declined [7]. Younger age at diagnosis (<60 years) was found to be predictive of poorer QOL across role, social, emotional, and cognitive functioning and on this basis it was concluded that longitudinal development of QOL was dependent upon age at diagnosis. Our team identified that baseline QOL was a strong predictor of QOL five years after treatment, with gender, private health insurance, social support, and threat appraisal influencing various QOL domains [8]. However, by comparison to the previous study [7] no effect was found for age. Hence, there is still lack of clarity about who is most at risk for poorer long term outcomes.

In this regard, optimism presents as a dispositional trait that influences both psychological and quality of life outcomes after cancer [9-11]. Optimism is defined as the generalized expectancy that more good things than bad will happen in the future [12]. The effect of optimism on outcomes appears to be mediated by threat appraisal [13], such that more optimistic people form more positive appraisals about the consequences of their cancer, and the likely outcomes, and from this experience less distress compared to people who are more pessimistic. Finally, social support is also an important and well accepted antecedent of both psychological and HR-QOL outcomes [9], such that the social environment in which a person lives may help (if positive) or hinder (if negative) the adjustment process.

In assessing QOL after cancer, two measurement approaches present: health-related quality of life and life satisfaction. Health-related quality of life (HR-QOL) is a multi-dimensional construct that incorporates, at minimum. the social, psychological and physical aspects of health [14]. On this view, these different aspects of HR-QOL make up layers of well-being that influence a person’s health status and that when disrupted by disease are expressed as decrements in these domains of quality of life. Accordingly, measurement approaches for HR-QOL tend to be domain and symptom-based, matching these layers. Over the past three decades a wealth of research, on a global scale, has emerged seeking to accurately measure HR-QOL, some of which has developed more generic approaches relevant to a person with any illness status [15], and some if which is tied to a specific disease, such as cancer and the various cancer types [16].

By contrast, life satisfaction is conceptualized as the outcome of a person’s judgment about the extent to which their current life quality matches their self-imposed life standards [17]. Hence, by contrast to the quite specific measurement approach used in HRQOL, assessment of life satisfaction is more global and relates more to internal individual standards that are likely, at least in part, dispositional. As an example, one person may perceive that if they were unable to function physically in a certain way that their life would be intolerable, whereas another person might view these functions as less crucial. The actual physical changes may be the same, however the judgment of what these changes mean differs, and hence so does the person’s overall life satisfaction.

Our study assessed the five year trajectories of both HR-QOL *and* life satisfaction in long term colorectal cancer survivors’ adjustment using an analytic approach known as growth mixture modelling [18]. Socio-demographic variables as well as optimism, threat appraisal and social support were included as predictor variables. In this approach we aimed to provide a more complete understanding of the QOL implications of colorectal cancer, both health-related and subjective, over time and more clearly identify what subgroups of patients are at risk for poorer outcomes.

## Materials and methods

### Participants and procedure

These data are from a longitudinal study of patients diagnosed with colorectal cancer (CRC), the sample and methods of which are described in detail elsewhere [19]. Ethical approval was provided by the University of Queensland. All residents in Queensland, Australia with a histologically confirmed diagnosis of a primary CRC between January 1, 2003, and December 31, 2004 were eligible. Eligibility criteria included speaking English; having no hearing, speech or cognitive disabilities that would prevent completing a telephone interview; and being aged between 20 and 80 years at diagnosis. The treating doctors of 3,626 eligible cases were approached in writing for permission to contact their patients regarding the study. The 3,182 cases for whom doctor consent was obtained were mailed a letter signed by their treating doctor explaining the study, a study information sheet and consent form. Those who did not respond were sent a second letter two weeks later. Following this, non-responders received two follow-up telephone calls to try and ascertain a response. Age, sex, tumor site and stage of disease were collected from pathology/medical records. Socio-demographic and medical variables were assessed by computer-assisted telephone interview at baseline; all other measures were by mailed self-report survey at each assessment point.

In all, 1966 participants provided informed consent and entered the study at 5-month after diagnosis (Time 1 (T1)). Consenting patients completed a self-administered questionnaire (SAQ) and computer assisted telephone interview (CATI). Participants were subsequently followed-up at 12 (T2), 24 (T3), 36 (T4), 48 (T5) and 60 months (T6) post-diagnosis. Among the 1884 participants who completed at least one SAQ, 450 participants (24%) had died by five years post-diagnosis (T6). We report data for these 1884 participants.

### Predictor variables

Socio-demographic and medical variables, including sex, age, education attainment, disease stage, cancer site, treatment type, number of comorbidities, the Accessibility/Remoteness Index of Australia (ARIA) [20], and the Index of Relative Socioeconomic Disadvantage (IRSD) [21] classification were assessed at baseline.

The ARIA represents the remoteness of residence when diagnosed with CRC and is based on road distance measurements. The IRSD is an area-based measure of socioeconomic status (SES) and considers factors such as the percentage of residents in each Statistical local areas (SLA: used as the geographic definition for area) on a low income, in unskilled occupations, and unemployed (among others). Its quintile represents the increasing advantage (Quintile 1, most disadvantaged).

Dispositional optimism was measured by the Revised Life Orientation Test [22], a 10-item scale consisting of six target items and four filler items. Items are scored on a five point responses scale where participants are asked to indicate their extent of agreement from 0 (strongly disagree) to 4 (strongly agree). Higher scores indicate greater optimism. Internal consistency for the current study was very good (α = 0.71-0.77). Cancer threat appraisal was measured by the Constructed Meaning Scale that assesses participants’ appraisal of the effect that cancer has had on their identity, relationship and perceived future. Participants indicate their agreement to eight items using a 4-point scale from 1 (strongly disagree) to 4 (strongly agree). Negatively worded items are reverse scored with response summed where a lower overall score indicates a more negative cognitive appraisal. Internal consistency for the current study was very good (α = 0.78-0.82). Perceived social support was measured using the Brief Social Support Questionnaire (SSQ-6) [23]. Participants rate how satisfied they are with various aspects of social support on a 5-point scale ranging from 1 (Very dissatisfied) to 5 (very satisfied). The scale of the SSQ-6 demonstrates high internal reliability (α = .92-.93) [23].

### Outcome variables

#### Satisfaction with life

The Satisfaction with Life Scale (SWLS) [24] was used to assess participant’s subjective cognitive well-being. The five item scale asks participants to indicate on 7-point scale ranging from 1 (Strongly disagree) to 7 (Strongly agree), with higher scores indicating a higher satisfaction with life. The SWLS demonstrates high internal reliability (α = .87) [24].

#### Health-related quality of life

Health-related quality of life was assessed using the Functional Assessment of Cancer Therapy-Colorectal (FACT-C) scale [16]. The FACT-C has 36 items in total and includes 5 subscales: physical (GP); functional (GF); social/family (GS); emotional well-being (GE) as well as a colorectal cancer specific domain (GC). Participants indicate how true the scale items have been for them in the past seven days on a scale from 0 (Not at all) to 4 (Very much). The FACT-C demonstrates good internal consistency (α = .85) [16].

### Statistical analyses

Growth mixture models (GMMs) [18,25] were applied to identify trajectory classes and predictors of membership in these classes, separately for the SWLS and the FACT-C scale and the FACT-C subscales. The GMMs are flexible to model individual growth trajectories from unobserved subpopulations (latent trajectory classes) with individual variation in growth parameters (such as intercept and slope) that are captured by random effects as continuous latent variables [26,27]. Another advantage of this analytical approach is that predictors of membership in the trajectory classes are identified within the GMM framework [28]. It implies that the “net” effect from each predictor can be quantified with the adjustment for the other predictors, and hence the unique characteristics for each trajectory class can be identified.

The GMM analyses were performed using Mplus Version 6.12 [28]. Non-linear GMMs (consisting of intercept, slope and quadratic growth parameters) were considered, where the within-class quadratic variance components were fixed at zero to avoid convergence problems and improper solutions [29]. Missing SWLS and FACT-C scores were handled in Mplus using a robust full information maximum likelihood (FIML) estimation procedure with the assumption that the missing scores are unrelated to the outcome variable; see Jung and Wickrama (2008) [29]. Estimates of covariance coverage for each pair of variables were checked for evaluating the impact of missing data on model convergence. Initial growth parameters were obtained by implementing a latent class growth analysis (assuming no within-class variance) fitted to the data. The GMM analyses were implemented with 200 random sets of starting values and 20 final optimizations. The number of trajectory classes *K* was determined using the Lo-Mendell-Rubin likelihood ratio test (LMR-LRT) statistic [29,30]. Finally, covariates were entered into the *K*-class (unconditional) GMM via multinomial logistic regression that compares the reference class (high SWLS or FACT-C scores) with the other trajectory classes (lower SWLS or FACT-C scores).

## Results

### Trajectory classes based on SWLS

**Figure 1:**
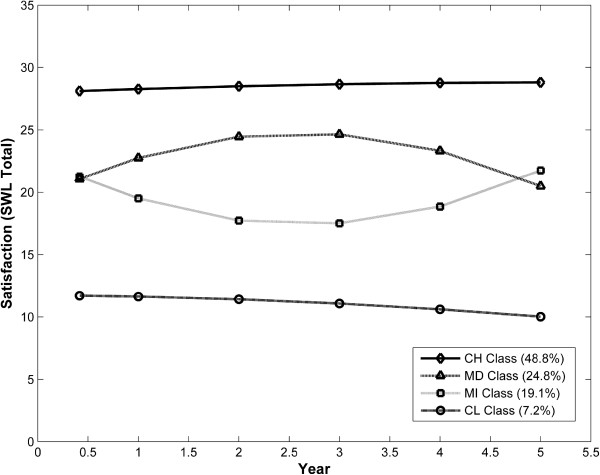
Estimated mean trajectories of SWLS (four-class model with 5 covariates, N = 1615).

Four distinct classes of trajectory patterns were identified for the SWLS using GMM (Figure 1). These classes were named based on the overall shape of the five year trajectory. The Constant High (CH) Satisfaction class indicates a group of patients (48.8%) who had a constantly high SWLS throughout the five years of follow-up period. The Medium Decrease (MD) class represents a group of patients (24.8%) whose SWLS rose gradually from a medium start and then at 3 years post-diagnosis began to decrease. The Medium Increase (MI) class (19.1% of patients) indicates a SWLS trajectory pattern which decreased steadily from a medium level and then increased at 3 years post-diagnosis. The Constant Low (CL) Satisfaction class represents a group of patients (7.2%) who had a constantly low SWLS.

Five predictors that significantly (p < 0.05) differentiated the SWLS trajectory classes with the Constant High (CH) as the comparison class were identified within the GMMs (Table 1). Compared to the Constant High (CH) class, patients with poor social support (adjusted OR = 1.44, 95% CI = 1.1 – 1.8), a more negative cognitive appraisal (adjusted OR = 1.14 per unit decrease in the appraisal score, 95% CI = 1.1 – 1.2) and those with low optimism (adjusted OR = 1.11 per unit decrease in the optimism score, 95% CI = 1.1 – 1.2) had an elevated chance of membership in the Medium Decrease (MD) class. Again, in comparison with the CH class, the Medium Increase (MI) class was characterized by younger age (adjusted OR = 1.25, 95% CI = 1.0 – 1.6), poor social support (adjusted OR = 1.62, 95% CI = 1.3 – 2.0), a more negative cognitive appraisal (adjusted OR = 1.16 per unit decrease in the appraisal score, 95% CI = 1.1 – 1.2) and low optimism (adjusted OR = 1.19 per unit decrease in the optimism score, 95% CI = 1.1 – 1.3), and less patients with less than eight years education (adjusted OR = 0.37, 95% CI = 0.2 – 0.9). Finally, compared to the CH class, the Constant Low (CL) Satisfaction class was differentiated by younger age (adjusted OR = 2.01, 95% CI = 1.5 – 2.8), poor social support (adjusted OR = 1.89, 95% CI = 1.4 – 2.5), a more negative cognitive appraisal (adjusted OR = 1.40 per unit decrease in the appraisal score, 95% CI = 1.3 – 1.5) and low optimism (adjusted OR = 1.31 per unit decrease in the optimism score, 95% CI = 1.2 – 1.4).

**Table 1:** Predictors of SWLS trajectory class membership (N = 1615)

**Predictor**	**Adjusted OR (95% CI) relative to constant high**			**p-value**^**c**^
	**Medium decrease**	**Medium increase**	**Constant low**	
Age (Younger^a^)	1.22 (1.0, 1.5)	1.25* (1.0, 1.6)	2.01* (1.5, 2.8)	< 0.001
Education level				0.010
Less than 8 years	1.04 (0.6, 1.9)	0.37* (0.2, 0.9)	0.26 (0.1, 2.2)	
8 – 12 years or College	Reference	Reference	Reference	
University	0.76 (0.4, 1.4)	0.60 (0.3, 1.1)	0.42 (0.1, 1.2)	
Social support (Dissatified^b^)	1.44* (1.1, 1.8)	1.62* (1.3, 2.0)	1.89* (1.4, 2.5)	< 0.001
Low cancer threat appraisal	1.14* (1.1, 1.2)	1.16* (1.1, 1.2)	1.40* (1.3, 1.5)	< 0.001
Low optimism	1.11* (1.1, 1.2)	1.19* (1.1, 1.3)	1.31* (1.2, 1.4)	< 0.001

* significant at 0.05 level on the adjusted log odds of being in the class versus the Constant High class.

^a^ patient age is categorized into “20-49”, “50-59”, “60-69”, “70-80” (treated as ordinal in GMMs).

^b^ satisfaction with social support received (ordinal scale from 0 “very dissatisfied” to 5 “very satisfied”).

^c^ likelihood ratio test (full model versus model without the predictor under consideration).

### Trajectory classes based on FACT-C scores

**Figure 2:**
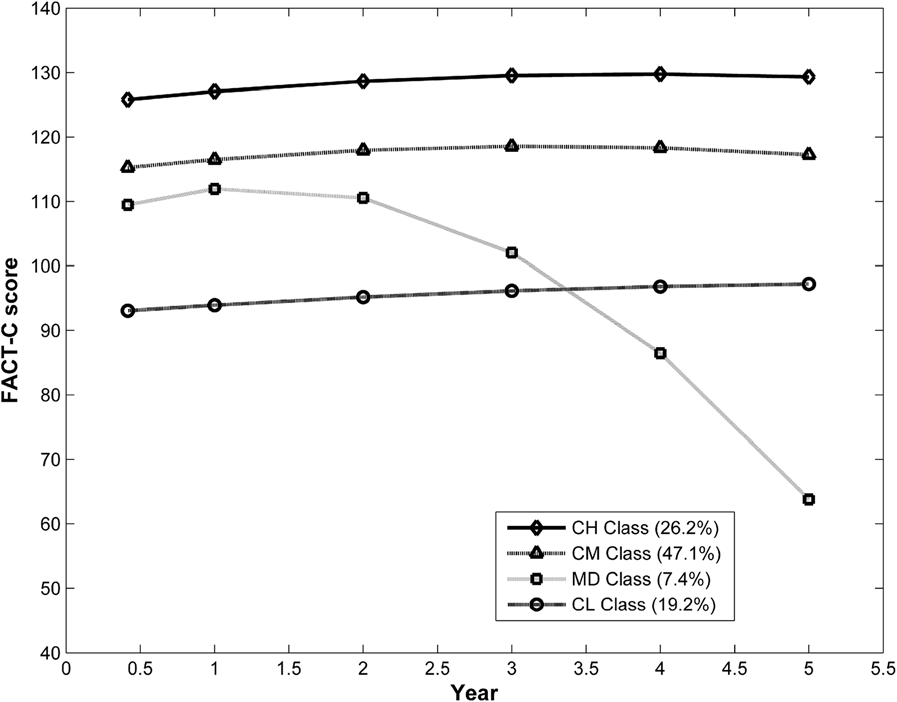
Estimated mean trajectories of FACT-C (four-class model with 6 covariates, N = 1618).

Four distinct classes of trajectory patterns for Quality of Life FACT-C scores were identified using GMM (Figure 2). The Constant High (CH) class indicates a group of patients (26.2%) who had a constantly high FACT-C throughout the five years of follow-up period. The Constant Medium (CM) class represents the largest group of patients (47.1%) whose FACT-C remained at a medium level throughout the follow-up period. The Medium Decrease (MD) class (7.4% of patients) indicates a FACT-C trajectory pattern which decreased dramatically from a medium level, especially after 2 years post-diagnosis. The Constant Low (CL) class represents a group of patients (19.2%) who had a constantly low FACT-C. Six predictors that with the Constant High (CH) Quality of Life as the comparison class significantly differentiated the trajectory classes are presented in Table 2. Unlike the findings for the SWLS Satisfaction scores in Table 1, education level was not a risk factor for differentiating the Quality of Life classes. However, we found that patients with poor social support had a higher chance of belonging to the Constant Medium (CM) class compared to the Constant High (CH) class (adjusted OR = 1.42, 95% CI = 1.0 – 2.0). Other significant predictors were negative cognitive appraisal (adjusted OR = 1.27 per unit decrease in the appraisal score, 95% CI = 1.2 – 1.4) and low optimism (adjusted OR = 1.10 per unit decrease in the optimism score, 95% CI = 1.0 – 1.2). Again compared to the Constant High (CH), the Medium Decrease (MD) class was characterized by patients with late disease stage (adjusted OR = 3.46, 95% CI = 1.7 – 7.1) and negative cognitive appraisal (adjusted OR = 1.24 per unit decrease in the appraisal score, 95% CI = 1.1 – 1.4). Finally, the Constant Low (CL) Quality of Life class was differentiated by female gender (adjusted OR = 2.09, 95% CI = 1.1 – 3.8), younger age (adjusted OR = 1.74, 95% CI = 1.3 – 2.4), late disease stage (adjusted OR = 2.13, 95% CI = 1.1 – 4.2), poor social support (adjusted OR = 2.32, 95% CI = 1.6 – 3.3), a more negative cognitive appraisal (adjusted OR = 1.77 per unit decrease in the appraisal score, 95% CI = 1.6 – 2.0), and low optimism (adjusted OR = 1.27 per unit decrease in the optimism score, 95% CI = 1.2 – 1.4).

**Table 2:** Predictors of FACT-C trajectory class membership (N = 1618)

**Predictor**	**Adjusted OR (95% CI) relative to constant high (CH) class**			**p-value**^**c**^
	**Constant medium**	**Medium decrease**	**Constant low**	
Sex (Male)	0.76 (0.5, 1.1)	1.55 (0.7, 3.6)	0.48* (0.3, 0.9)	0.024
Age (Younger^a^)	0.99 (0.8, 1.3)	1.22 (0.8, 1.9)	1.74* (1.3, 2.4)	< 0.001
Disease stage				0.002
Stages 0, I, II	Reference	Reference	Reference	
Stages III, IV	1.26 (0.8, 2.0)	3.46* (1.7, 7.1)	2.13* (1.1, 4.2)	
Unknown	0.74 (0.4, 1.4)	1.21 (0.4, 3.4)	0.69 (0.3, 1.6)	
Social support (Dissatified^b^)	1.42* (1.0, 2.0)	1.14 (0.7, 1.9)	2.32* (1.6, 3.3)	< 0.001
Low cancer threat appraisal	1.27* (1.2, 1.4)	1.24* (1.1, 1.4)	1.77* (1.6, 2.0)	< 0.001
Low optimism	1.10* (1.0, 1.2)	1.06 (1.0, 1.1)	1.27* (1.2, 1.4)	< 0.001

* significant at 0.05 level on the adjusted log odds of being in the class versus the Constant High class.

^a^ patient age is categorized into “20-49”, “50-59”, “60-69”, “70-80” (treated as ordinal in GMMs).

^b^ satisfaction with social support received (ordinal scale from 0 “very dissatisfied” to 5 “very satisfied”).

^c^ likelihood ratio test (full model versus model without the predictor under consideration).

### Trajectory classes based on five FACT-C subscales

Distinct classes of trajectory patterns for FACT-C GP, GS, GE, GF and C subscales were identified using GMM. Predictors that significantly differentiated the trajectory classes were also found to be different between the five FACT-C subscales. In Table 3, we present the adjusted ORs of significant predictors for comparing the constant low (CL) or low decrease (LD) classes relative to the constant high (CH) class, with the five subscales. The CL and CH trajectories have a constantly low and high score throughout the follow-up period, respectively. The LD trajectory decreases from an early low start.

**Table 3:** Predictors of CL trajectory class for various FACT-C sub domains (N = 1618)

**Predictor**	**Adjusted OR (95% CI) for constant low (CL) class relative to constant high (CH) class**				
	**Physical**	**Social/Family**	**Emotional wellbeing**	**Functional**	**Colorectal cancer specific**
Sex (Male)		3.52* (1.9, 6.4)		0.39* (0.2, 0.8)	0.23* (0.1, 0.4)
Age (Younger^a^)		1.48* (1.1, 2.1)	1.89* (1.3, 2.7)		
Socioeconomic Disadv.					1.37* (1.1, 1.8)
Disease stage					
Stages 0, I, II	Reference		Reference	Reference	
Stages III, IV	2.61* (1.7, 3.9)		2.20* (1.2, 3.9)	2.97* (1.5, 5.9)	
Unknown					
Remoteness of residence					
Major city					0.42* (0.2, 0.9)
Inner regional					Reference
Outer regional					
Remote/very remote					
Social support (Dissatified^b^)		7.83* (3.7, 16)	1.29* (1.0, 1.6)	1.95* (1.3, 2.9)	1.51* (1.2, 2.0)
Low cancer threat appraisal	1.09* (1.0, 1.2)	1.19* (1.1, 1.3)	1.29* (1.1, 1.5)	1.79* (1.5, 2.1)	1.37* (1.2, 1.5)
Low optimism	1.06* (1.0, 1.1)	1.26* (1.2, 1.4)	1.22* (1.1, 1.3)	1.26* (1.1, 1.5)	1.14* (1.1, 1.2)

* shown only significant results at 0.05 level on the adjusted log odds of being in the CL class versus the CH class.

^a^ patient age is categorized into “20-49”, “50-59”, “60-69”, “70-80” (treated as ordinal in GMMs).

^b^ satisfaction with social support received (ordinal scale from 0 “very dissatisfied” to 5 “very satisfied”).

We found that poor social support, lower cognitive appraisal and optimism significantly increased the chance of lower scores in all Quality of Life FACT-C subscales (except the GP subscale for social support). Gender significantly differentiated the CL/LD class from the CH class for the FACT-C GS, GF and C subscales, while a younger age significantly increased the chance of lower scores in GS and GE subscales. Late disease stage also increased the chance of belonging to the CL/LD class, compared to the CH class, for the GP, GE and GF subscales. The C subscale appears to be distinct among the five FACT-C subscales, because there were two unique predictors (socioeconomic disadvantage and remoteness of residence) that significantly differentiated the CL/LD class from the CH class.

## Discussion

Three quarters of all CRC survivors experienced consistently medium to high HR-QOL for the five years after their cancer diagnosis; and one in five had consistently poorer outcomes with a smaller group declining in HR-QOL at three years. Hence, it appears that while many CRC survivors do well over time, a sizeable subgroup carry a substantive long term health burden. Characteristics that placed individuals at risk of these negative outcomes were clear: poorer psychological resources predicted a more negative long term trajectory of QOL for CRC survivors. This was the case for the person’s subjective judgment of their QOL, as well as their HR-QOL; and suggests that in order to improve these outcomes, interventions early in the illness experience to improve these resources are needed. In this context, interventions based on social cognitive theory may be most salient [31]. Therapy targets here include self-efficacy [32], outcome expectations [33], and self-regulation [32], with meta-analyses suggesting strategies that target these components are associated with better outcomes [31]. Support interventions that promote optimism and hope in a supportive group or dyadic peer setting may also be indicated, where peer interaction acts as the mechanism for change through the processes of social comparison and social support [34].

Younger age was confirmed also as a risk factor for poorer QOL outcomes, a finding that is consistent with other cancer types and likely related to life stage demands and expectations [35,36]. Specifically, younger CRC survivors may still be building careers and families, have greater financial responsibilities, and also see their cancer as occurring out of age-related usual expectations for health and illness events. A novel finding was that remoteness of residence and socioeconomic disadvantage uniquely predicted poorer outcomes for the colorectal specific quality of life domain that reflects disease-specific iatrogenic effects. This seems consistent with findings that increasing remoteness of residence and area-based social disadvantage are independently associated with lower colorectal cancer survival [37]. The reasons for this are unclear, however, evidence that patients living outside major cities have a higher risk of advanced colorectal cancer at diagnosis[38] and also that survival decreases with increasing distance to the patient’s closest radiotherapy treatment facility [39] suggest that differential access to diagnostic and treatment services are possible factors. Hence, these patients may experience greater disease burden as a result of poorer access to optimal medical treatment.

While the patterns for prediction for life satisfaction were similar to that for HR-QOL, there were some substantive differences. First, almost half of all participants reported constant high life satisfaction. Specifically, for these individuals the experience of having cancer did not lead them to judge their lives globally as being impaired or less than ideal, based on their own values or internal standards. For those participants whose life satisfaction trajectories varied over time (both declining and increasing) they seemed to return largely to their baseline state at five years. This suggests two conclusions. First, that while life satisfaction may respond to external events changes (such as a cancer diagnosis), it is a temporally stable phenomenon [40]. This finding adds to the body of knowledge about our understanding of life satisfaction as a construct. Second, this may also speak to individuals’ resilience to cancer. Resilience is broadly conceptualized as the ability to sustain trauma without developing reactive psychopathology [41]. Previous research comparing cancer survivors to an age, gender and education matched sample, concluded that while survivors do experience impaired psychological functioning in some domains, such as social wellbeing, they are resilient [42]. The ability of cancer survivors to maintain a stable sense of life satisfaction after their cancer experience may also, at least in part, reflect resilience.

In this regard, a limitation of the present study is that we did not specifically measure resilience such that our picture of what factors matters most in predicting quality of life outcomes may be incomplete. This is an area for future research. As well, although we were able to recruit a substantive study sample it is possible that those who did not participate varied in important ways, for example may have been more distressed or less health literate. As we did not have data on non-respondents we were unable to assess this. However, key strengths of this study include the prospective design with long term follow up, application of well validated and reliable measures, and a large population based sample.

In conclusion, the present results add to the growing body of knowledge about the heterogeneous nature of individual adjustment after cancer and further highlight the importance of considering inter-individual differences in research with this population group, as well as in planning service delivery. The contrast between trajectory patterns for HR-QOL and life satisfaction suggests that they are distinct adjustment outcomes. Life satisfaction appears in this population group to be temporally stable and this may reflect individuals’ psychological resilience to the experience of cancer. There is growing recognition of the importance of patient-focused outcomes in cancer care [43]; where the quality of a person’s life and the personal preferences and values of that person guide their health care. The inclusion of life satisfaction in future research as a distinct adjustment outcome based on the individual’s point of reference is warranted.

## Competing interests

Authors’ disclosures of potential conflicts of interest: The author(s) indicated no potential conflicts of interest.

## Authors’ contributions

JD conceived the project, participated in its design and coordination and led manuscript development; SKN performed the statistical analysis and guided interpretation of results; PB JA and PY participated in its design and coordination, provided methodological guidance and helped to draft the manuscript: WB provided direction towards analyses and intellectual comment for interpretation of results; SKC directed the study, its design and coordination, and helped to draft the manuscript. All authors read and approved the final manuscript.
